# Aon: a service to augment Alliance Genome Resource data with additional species

**DOI:** 10.1186/s13104-023-06577-8

**Published:** 2023-10-27

**Authors:** Sophie K. Kearney, Alexander Berger, Erich Baker

**Affiliations:** 1 Department of Computer Science, Baylor University, One Bear Place Box 97356, Waco, 76798 USA; 2 The Jackson Laboratory, 600 Main St., Bar Harbor, 24105 USA

**Keywords:** Ortholog, Homology, AGR

## Abstract

**Objective:**

Cross-species comparative genomics requires access to accurate homology data across the entire range of annotated genes. The Alliance of Genome Resources (AGR) provides an open-source and comprehensive database of homology data calculated using a wide array of algorithms at differing stringencies to elucidate orthologous relationships. However, the current AGR application program interface (API) is limited to five homology endpoints for nine species. While AGR provides a robust resource for several canonical species, its utility can be greatly enhanced by increased filtering and data processing options and incorporating additional species.

**Results:**

Here, we describe a novel API tool, AON, that expands access to the AGR orthology resource by creating a data structure that supports 50 additional endpoints. More importantly, it provides users with a framework for adding bespoke endpoints, custom species, and additional orthology data. We demonstrate AON’s functionality by incorporating the service into the GeneWeaver ecosystem for supporting cross-species data analysis.

## Introduction

A well-defined mapping of homologous genes between species is essential for effective comparative genomic analysis [1] in an effort to enhance our understanding of shared biological processes and disease mechanisms [2]. Accurate homology data also improves annotations of newly sequenced genomes [3], helps to create and understand phylogenic relationships, and facilitates a deeper appreciation of the impact of single variant mutations on larger systems [4].

The Alliance of Genome Resources (AGR) provides a collection of gene-centric data for nine essential model organisms [5]. Included in AGR’s publicly-available data set is a compilation of homology data derived from multiple sources. Homology data uses a range of algorithms [6–17] and reports differing scoring stringencies to elucidate orthologous relationships across a range of confidences [5]. The diverse background of orthology data and associated metadata allows for a deeper understanding of functional genomics across species.

AGR has provided application program interface (API) access to facilitate interactions within a limited range of species: *Mus musculus*, *Rattus norvegicus*, *Saccharomyces cerevisiae*, *Caenorhabditis elegans*, *Drosophila melanogaster*, *Danio rerio*, *Homo sapiens*, *Xenopus laevis*, and *Xenopus tropicalis*. While these nine species represent a core of useful model organisms, there is no existing mechanism to add additional species as needed. Furthermore, the utility of the existing API to programmatically navigate homology data is limited by a small number of available endpoints [5], specific URLS to manipulate or obtain data from a database through HTTP requests, and necessarily requires that users are computationally savvy enough to search for, modify, and incorporate data sets into custom projects.

GeneWeaver (https://www.geneweaver.org/) is a public repository of gene-centric data with accompanying web-based tools for comparative genomic analysis across multiple species [18]. Until recently, GeneWeaver has relied on internal static mapping approaches to facilitate cross-species alignment. We demonstrate here an augmentation of AGR API services that allows for complex homology comparisons while allowing for the addition of custom species, called the AGR Orthology Normalizer (AON). To demonstrate the robustness of our approach, we describe how the augmented service was incorporated into the larger GeneWeaver ecosystem.

## Main text

AON is a novel service that accesses AGR data through 50 unique endpoints. Descriptions of each endpoint can be found in the software documentation (https://bitbucket.org/sophie_kearney1/aon/src/master/Endpoints_Table.pdf). Our software allows for the incorporation of data from additional reference sources to augment accuracy models and add alternative species. Adding these endpoints increases the functionality of the dataset by providing complete access to all of the data as well as individualized endpoints for each table that can be further specified by the user’s parameter choice. This level of flexibility empowers uses to tailor their data queries to exactly fit their research needs.

### Materials and methods

#### Software requirements

AON uses the Python (3.11.1) Flask (2.2.2) web framework to serve and manage endpoints accompanied by a PostgreSQL (14.2) database back-end. Associated required Python packages are listed in the software documentation (https://bitbucket.org/sophie_kearney1/aon/src/master/aon-service/requirements.txt).

#### Local database management

**Fig. 1:**
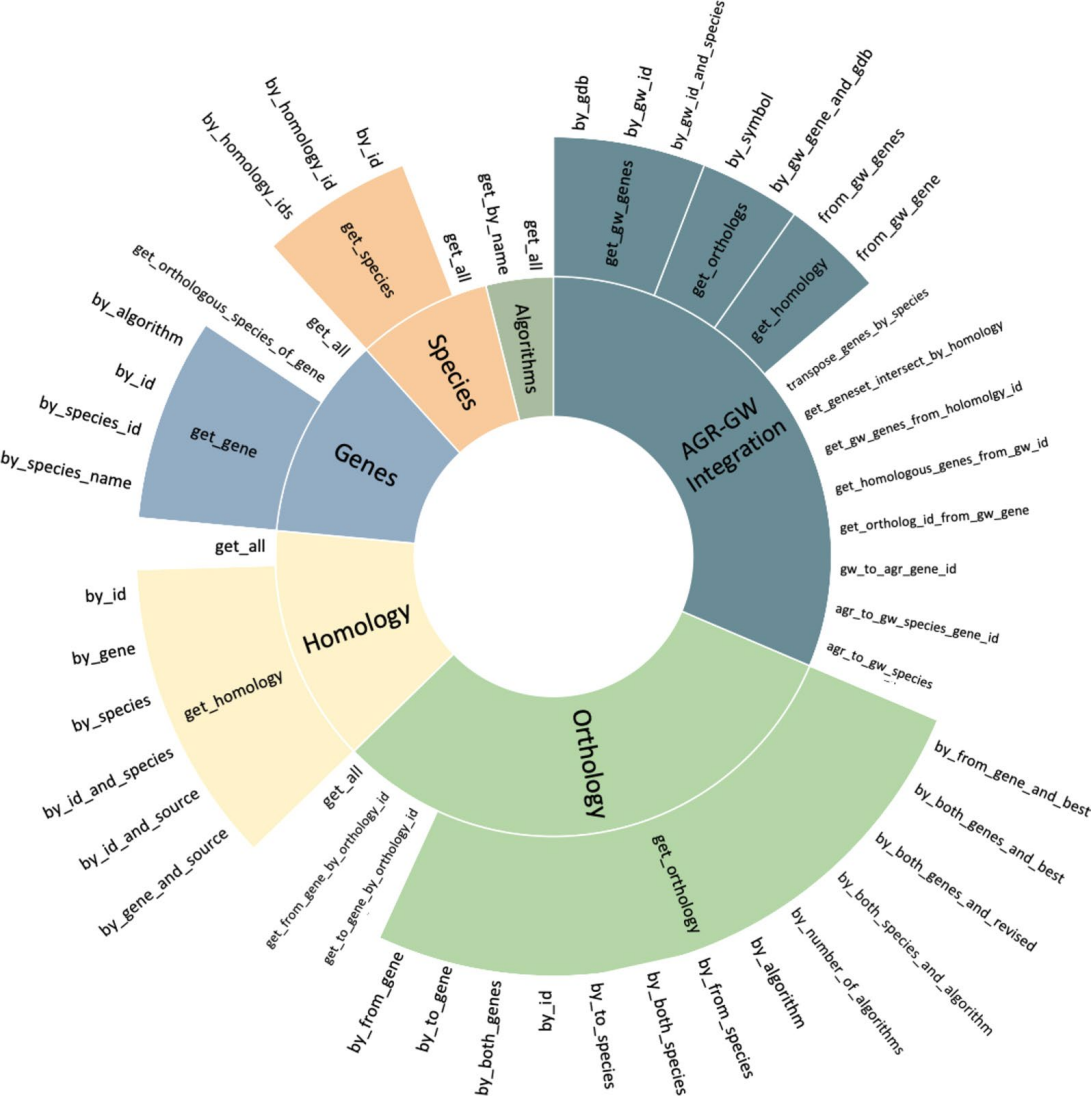
**Additional endpoints.** There are six categories of novel endpoints included in this service. The Genes, Species, Algorithms, Homology, and Orthology categories include endpoints from corresponding tables in AON database. The AGR-GW Integration category includes endpoints used specifically for integration of AON service with GeneWeaver and utilizes data from GeneWeaver. The subcategories represent endpoints that return similar output but have differing inputs. For example, within the Genes category, the get_gene subcategory indicates that genes can be queried by_algorithm, by_id, by_species_id, and by_species_name

A set of AON scripts manage the process of creating and populating unique PostgreSQL data tables required to run the local service. Build scripts retrieve orthology and gene data from the canonical AGR source as a tab-separated value (TSV) file (https://www.alliancegenome.org/downloads#orthology) and load all the appropriate data into local database tables [5]. These tables include a highly normalized structure to support AON custom queries and represent tables for genes, species, algorithms, and homologies. If users wish to implement endpoints that integrate with GeneWeaver resources and GeneWeaver’s extensive repository of gene sets, GeneWeaver tables are required. Specifically, the *gene*, *species*, and *gene database (genedb)* tables are necessary to translate GeneWeaver species and identifiers. GeneWeaver tables can be reconstituted from comma-separated value (CSV) files located in AON repository. The AON repository also contains Python scripts to automate the loading of these tables from the provided CSV files into the user’s SQL database. The resulting service provides 50 novel endpoints to query whole tables, multiple parameters, identifier mapping between AGR and GeneWeaver databases, and integration of the AON service into GeneWeaver, as seen in Fig. 1.

#### Incorporating missing species

**Fig. 2:**
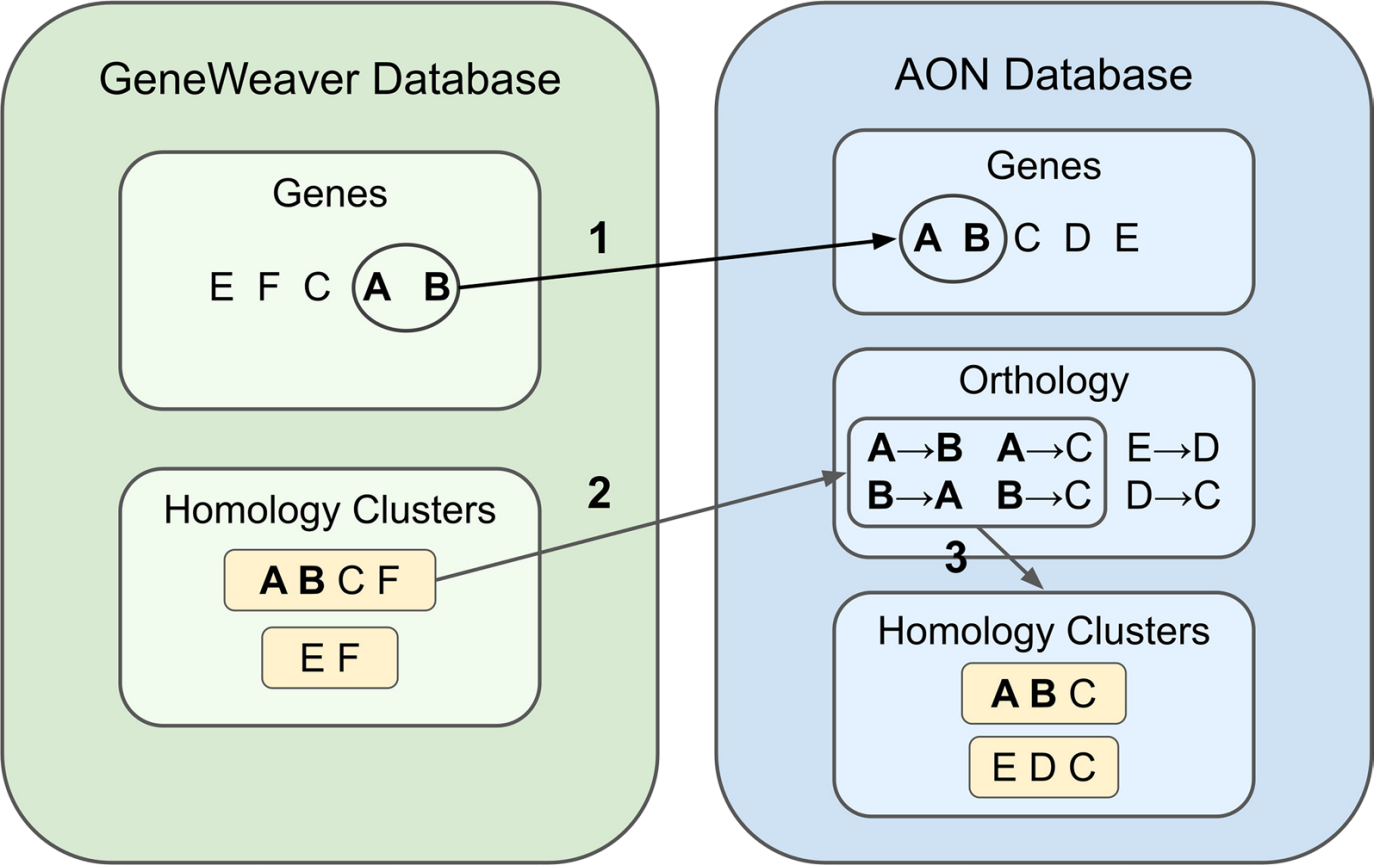
**Augmenting native AGR homology data with additional species.** Homology data from additional species may be added to existing AGR-supplied data in the local environment. This figure illustrates generalized mapping from additional resources. For example, if (1) genes A and B are derived from a missing species, they are added to AON gene table. (2) Pairwise homology information is simultaneously added to the AGR orthology table. In this example, GeneWeaver homology clusters are decomposed into their constitutive pairwise relationships. If a gene is not represented in AGR, it is not added (ex: F) in the pairwise orthology table. Relationships are not added if they connect two genes where neither is from the missing species. (3) For producing endpoints that integrate into GeneWeaver, pairwise relationships are aggregated into homology clusters

Current AON functionality supports adding missing species and their associated genes and orthologous relationships. This can be done manually or from existing mappings found within GeneWeaver. For example, associating GeneWeaver data tables will provide information for *Macaca mulatta*, *Gallus gallus*, and *Canis familiaris*. For new species, scripts parse GeneWeaver-associated gene objects required for the AON *gene* table and convert appropriate identifiers. Simultaneously, homology clusters are separated into pairwise orthologous relationships to map genes from newly added species to genes from existing species, see Fig. 2. To avoid false positives, only mappings from new species are added. Checks are included to remove duplicates.

#### GeneWeaver integration

GeneWeaver homology mapping relies on the creation of homology clusters. Consequently, AON creates a homology table to manage the construction of homology clusters from pairwise relationships. AON scripts iterate through the orthology table to sort missing genes into appropriate existing clusters, adding new clusters when necessary.

### Results

#### Service usage

The novel AON service provides 50 API endpoints that may be evaluated independently or incorporated into other software projects. Endpoints build on existing AGR functions to provide more robust access to harmonized orthology data from existing AGR species or new, user-defined species. The service is extensible, allowing users to add additional endpoints, and data is returned in JSON format for rapid cross-platform usage. While AON provides a host of scripts to articulate with the GeneWeaver web service, GeneWeaver interactions are not required for stand-alone implementations of AON.

#### Adding additional species

**Fig. 3:**
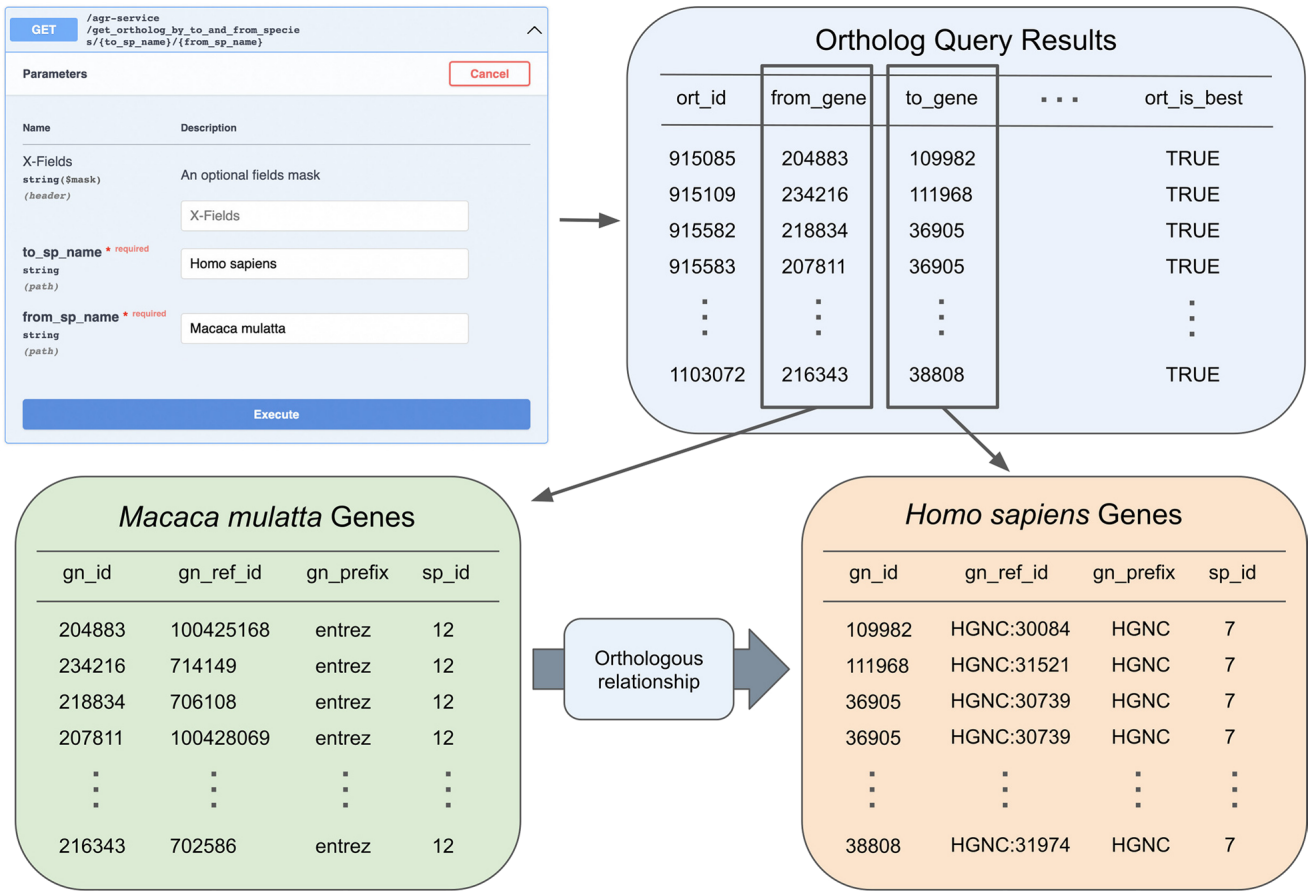
**Example endpoint to retrieve orthologous genes.** Here we demonstrate the endpoint get_ortholog_by_to_and_from_species, in which all orthologs from an added species, *Macaca mulatta*, to an existing AGR species, *Homo sapiens*, are retrieved by AON. To do this, AON translates the species name into species ID numbers, retrieves all genes from each species, and queries the ortholog table with these genes, from *Macaca mulatta* to *Homo sapiens*. The result is a subset of the ortholog table containing relationships between these two species. The genes identified by these relationships are orthologous. This figure illustrates how the software can transition between different accession numbers, Entrez accession IDs for *Macaca mulatta* and species-specific HGNC for *Homo sapiens*

AON was designed to rapidly ingest additional species for orthology mapping. Here, we demonstrate this functionality by providing scripts that support species that are mapped by GeneWeaver but not AGR. Each new species (*Gallus gallus*, *Canis familiaris*, and *Macaca mulatta*) was registered in the AON species table and their individual gene identifiers were added to the AON gene table. The service accepts NCBI, Ensembl, or other species-specific accession numbers. For example, the service can find orthologous relationships between data from Entrez and HGNC, as seen in Fig. 3. With the three added species, *Gallus gallus*, *Canis familiaris*, and *Macaca mulatta*, the service contains species-specific accession numbers from Xenbase, MGI, Entrez, HGNC, ZFIN, Ensembl, FB, WB, SGD, RGD, and CGNC. Since the new species are not in AGR, homology mapping from GeneWeaver is used to infer pairwise mapping. However, any user-provided gene mapping is acceptable.

### Discussion

AGR’s novel publicly-available repository of multi-species genomic data allows users to access aggregated data sources in a standardized format. Rigorous quality control measures ensure that repository data is up to date and accurately reflects the primary source material. AGR orthologous mappings remove user burden to define orthologs, provide graded quality metrics, and act as a powerful tool in understanding cross-species genomic relationships. AGR has created several tools allowing users to extract significant findings from their database, such as an API that allows software to securely interact with the most current version of the AGR database.

AON builds upon the potential of the AGR database and adds capabilities to the current AGR API. Specifically, AON increases the availability and granularity of AGR orthology data access, while adding the ability to incorporate novel species. Figure 3 demonstrates using AON to identify orthologous genes between an existing AGR species and a newly added species. To demonstrate the utility of the new service, we implemented it within the well-known GeneWeaver framework to allow AGR-driven orthology mapping in real time with additional species data. AON provides greater access to AGR orthology data by providing scalability to the number of community generated endpoints and the incorporation of additional species.

## Data Availability

AGR Orthology data can be downloaded at https://www.alliancegenome.org/downloads#orthology. AON can be accessed at https://bitbucket.org/sophie_kearney1/aon/src/master/.

## References

[1] Zdobnov EM, Kuznetsov D, Tegenfeldt F, Manni M, Berkeley M, Kriventseva EV (2021). OrthoDB in 2020: evolutionary and functional annotations of orthologs. Nucleic Acids Res.

[2] Fang G, Bhardwaj N, Robilotto R, Gerstein MB (2010). Getting started in gene orthology and functional analysis. PLoS Comput Biol.

[3] Wilson CA, Kreychman J, Gerstein M (2000). Assessing annotation transfer for genomics: quantifying the relations between protein sequence, structure and function through traditional and probabilistic scores. J Mol Biol.

[4] van der Heijden RTJM, Snel B, van Noort V, Huynen MA (2007). Orthology prediction at scalable resolution by phylogenetic tree analysis. BMC Bioinform.

[5] The Alliance of Genome Resources Consortium (2020). Alliance of Genome Resources Portal: unified model organism research platform. Nucleic Acids Res..

[6] Thomas PD, Ebert D, Muruganujan A, Mushayahama T, Albou L-P, Mi H (2022). PANTHER: making genome-scale phylogenetics accessible to all. Protein Sci..

[7] Bradford YM, Van Slyke CE, Ruzicka L, Singer A, Eagle A, Fashena D, Howe DG, Frazer K, Martin R, Paddock H, Pich C, Ramachandran S, Westerfield M (2022). Zebrafish information network, the knowledgebase for *Danio rerio* research. Genetics..

[8] Fuentes D, Molina M, Chorostecki U, Capella-Gutiérrez S, Marcet-Houben M, Gabaldón T (2022). PhylomeDB v5: an expanding repository for genome-wide catalogues of annotated gene phylogenies. Nucleic Acids Res..

[9] Cunningham F, Allen JE, Allen J, Alvarez-Jarreta J, Amode M, Armean I, Austine-Orimoloye O, Azov A, Barnes I, Bennett R, Berry A, Bhai J, Bignell A, Billis K, Boddu S, Brooks L, Charkhchi M, Cummins C, Da Rin Fioretto L, Davidson C, Dodiya K, Donaldson S, El Houdaigui B, El Naboulsi T, Fatima R, Giron CG, Genez T, Martinez J, Guijarro-Clarke C, Gymer A, Hardy M, Hollis Z, Hourlier T, Hunt T, Juettemann T, Kaikala V, Kay M, Lavidas I, Le T, Lemos D, Marugán JC, Mohanan S, Mushtaq A, Naven M, Ogeh D, Parker A, Parton A, Perry M, Piližota I, Prosovetskaia I, Sakthivel M, Salam A, Schmitt B, Schuilenburg H, Sheppard D, Pérez-Silva J, Stark W, Steed E, Sutinen K, Sukumaran R, Sumathipala D, Suner M-M, Szpak M, Thormann A, Tricomi FF, Urbina-Gómez D, Veidenberg A, Walsh T, Walts B, Willhoft N, Winterbottom A, Wass E, Chakiachvili M, Flint B, Frankish A, Giorgetti S, Haggerty L, Hunt S, IIsley G, Loveland J, Martin F, Moore B, Mudge J, Muffato M, Perry E, Ruffier M, Tate J, Thybert D, Trevanion S, Dyer S, Harrison P, Howe K, Yates A, Zerbino D, Flicek P. Ensembl. 2022;50, 988–995. 10.1093/nar/gkab1049.

[10] Emms DM, Kelly S (2019). OrthoFinder: phylogenetic orthology inference for comparative genomics. Genome Biol..

[11] Sonnhammer ELL, Ostlund G (2015). InParanoid 8: orthology analysis between 273 proteomes, mostly eukaryotic. Nucleic Acids Res..

[12] DeLuca TF, Cui J, Jung J-Y, St Gabriel KC, Wall DP (2012). Roundup 2.0: enabling comparative genomics for over 1800 genomes. Bioinformatics..

[13] Nevers Y, Kress A, Defosset A, Ripp R, Linard B, Thompson JD, Poch O, Lecompte O (2019). OrthoInspector 3.0: open portal for comparative genomics. Nucleic Acids Res..

[14] Altenhoff AM, Train C-M, Gilbert KJ, Mediratta I, Mendes de Farias T, Moi D, Nevers Y, Radoykova H-S, Rossier V, Warwick Vesztrocy A, Glover NM, Dessimoz C (2021). OMA orthology in 2021: website overhaul, conserved isoforms, ancestral gene order and more. Nucleic Acids Res..

[15] Kaduk M, Riegler C, Lemp O, Sonnhammer ELL (2016). HieranoiDB: a database of orthologs inferred by hieranoid. Nucleic Acids Res..

[16] Seal RL, Braschi B, Gray K, Jones TEM, Tweedie S, Haim-Vilmovsky L, Bruford EA (2003). Genenames.org: the HGNC resources in 2023. Nucleic Acids Res..

[17] Schreiber F, Patricio M, Muffato M, Pignatelli M, Bateman A (2014). TreeFam v9: a new website, more species and orthology-on-the-fly. Nucleic Acids Res..

[18] Baker EJ, Jay JJ, Bubier JA, Langston MA, Chesler EJ (2012). GeneWeaver: a web-based system for integrative functional genomics. Nucleic Acids Res.

